# Autophagosome biogenesis and human health

**DOI:** 10.1038/s41421-020-0166-y

**Published:** 2020-06-02

**Authors:** Tsuyoshi Kawabata, Tamotsu Yoshimori

**Affiliations:** 1 Department of Stem Cell Biology, Atomic Bomb Disease Institute, Nagasaki University, Nagasaki, 852-8523 Japan; 2 Department of Genetics, Graduate School of Medicine, Osaka University, Osaka, 565-0871 Japan; 3 Department of Intracellular Membrane Dynamics, Graduate School of Frontier Biosciences, Osaka University, Osaka, 565-0871 Japan

**Keywords:** Protein quality control, Macroautophagy

## Abstract

Autophagy degrades the cytoplasmic contents engulfed by autophagosomes. Besides providing energy and building blocks during starvation via random degradation, autophagy selectively targets cytotoxic components to prevent a wide range of diseases. This preventive activity of autophagy is supported by many studies using animal models and reports identifying several mutations in autophagy-related genes that are associated with human genetic disorders, which have been published in the past decade. Here, we summarize the molecular mechanisms of autophagosome biogenesis involving the proteins responsible for these genetic disorders, demonstrating a role for autophagy in human health. These findings will help elucidate the underlying mechanisms of autophagy-related diseases and develop future medications.

## Introduction

Macroautophagy (hereafter autophagy) is a bulk intracellular degradation system that digests self-components, as its name is derived from the Greek words auto (self) and phagy (eating). Autophagy degrades the cytoplasmic contents engulfed by a characteristic double-membrane structure called an autophagosome, which is ~1 µm^1^. This nonselective degradation system is essential for survival during starved conditions as it provides cells with buck-up energy and building blocks required to retain cellular functions. Moreover, a low level of basal autophagy constitutively maintains the turnover of self-components even without any exogenous stresses. Furthermore, autophagy has been shown to target invading bacteria in a highly specific manner to support the viability of host cells, suggesting that autophagy can selectively degrade harmful substances that cannot be digested by other pathways such as proteasomal degradation pathway. The selective autophagy eliminates damaged organelles, such as mitochondria and lysosomes or protein aggregates, that play a causative role in a range of diseases, including neurodegeneration. Recent studies have shown that the number of the type of targets is larger than previously anticipated, calling attention to a role of autophagy as a biological defense mechanism^2^. Although autophagosome observation by electron microscope and biochemical analysis had been suggested for basic topology and workflow of autophagy since the 1960s, the underlying molecular mechanisms were not elucidated for many years due to the difficulty of analysis. Ohsumi et al. carried out a large-scale genetic screening of *Saccharomyces cerevisiae* mutants and identified a series of genes essential for autophagy, some of which are overlapped with genes independently identified by Thumm’s lab and Klionsky’s lab^3^. These results led to powerful genetic analyses and striking mechanistic findings and an establishment of basic mechanism of autophagy. These reports were followed by subsequent research on autophagy in mammals, which led to the identification of the role for autophagy in human health; this discovery was awarded the 2016 Nobel Prize in Physiology or Medicine.

Our current understanding of autophagy in physiology is strongly supported by studies using model experimental systems. Moreover, many human hereditary diseases caused by autophagy-related genes have been reported in the last decade, contributing to the elucidation of the role of autophagy in human physiology. In this paper, we summarize the molecular mechanism of mammalian autophagy and describe the current understanding of human hereditary disorders to elucidate the physiological significance of autophagy in humans for future studies. Although not mentioned in detail here, microautophagy, which directly delivers substrates into vacuoles or lysosomes, and chaperone-mediated autophagy (CMA), which transports substrates to lysosomes via activity of chaperones, are also involved in maintenance of cellular homeostasis^4^.

### Molecular mechanism of autophagosome biogenesis

**Fig. 1:**
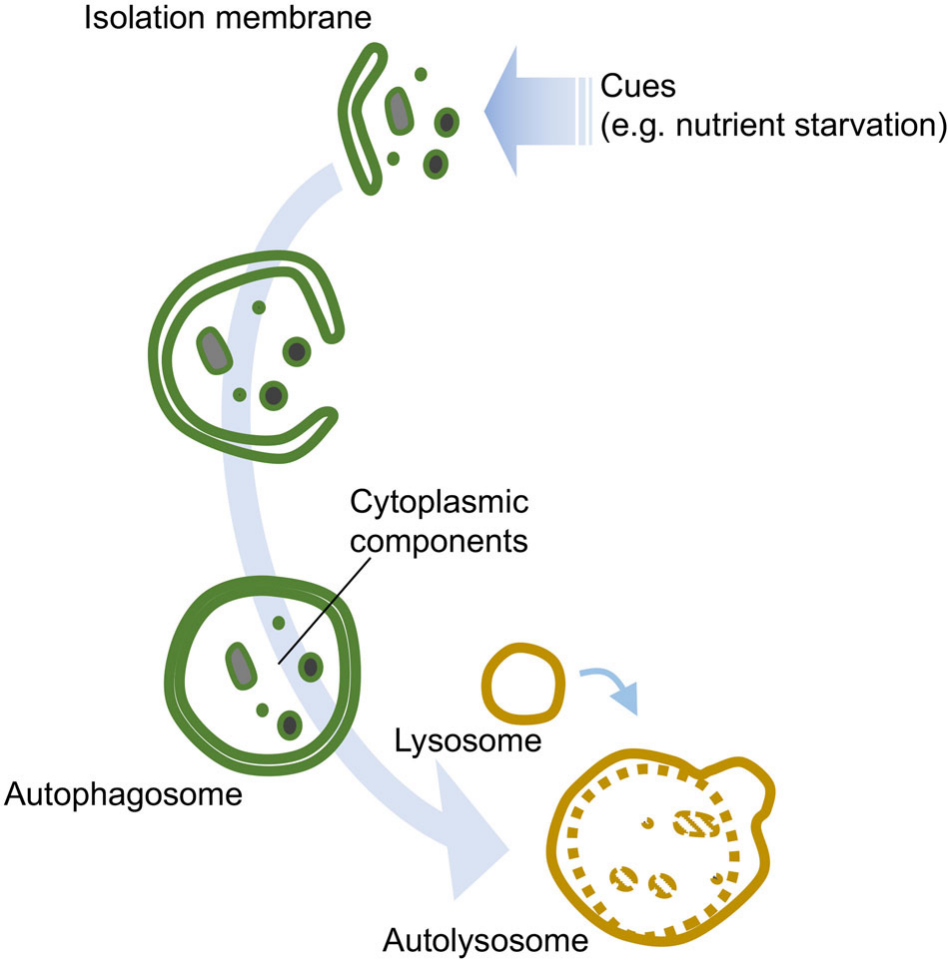
A brief overview of autophagy. Upon cues such as a starvation signal, isolation membranes are generated de novo that extend to form autophagosomes sequestering cytoplasmic components. The contents are further digested by fusion with lysosomes containing a variety of hydrolases.

The most characteristic feature of autophagy is the engulfment of cytoplasmic components by a double-membrane structure called an autophagosome. Unlike other organelles such as mitochondria, autophagosomes are generated de novo upon induction by stresses such as starvation. First, an isolation membrane (phagophore) is generated at the autophagosome formation site, followed by elongation and closure of the edge of the membranes to form autophagosomes. By the subsequent fusion with lysosomes containing various hydrolases, autophagosomes mature to autolysosomes so that the contents with the inner membranes are degraded (Fig. 1). In an electron micrograph, autophagosomes appear as a double-membrane structure with a cytoplasmic fraction, and autolysosomes have a single-membrane structure with a high electron density. This dynamic process of degradation is carried out by several functional units consisting of autophagy-related proteins. Following the identification of the yeast autophagy-related gene group ATG, researchers have found that homologs of these genes exist in mammals and are functionally conserved (Table 1). These proteins are mainly divided into groups with four different functions: the ULK1 protein kinase complex, the transmembrane protein ATG9 in single-membrane vesicles called ATG9 vesicles, the phosphoinositide 3-kinase (PI3K) complex, and a series of proteins that carry out post-translational modification of Atg8 homologs. The ULK1 complex and ATG9 vesicles are required for the formation of isolation membranes, and the PI3K complex is needed for the recruitment of factors required for the modification of the downstream Atg8 homologs to the isolation membrane. Atg8 homologs play a role in coordinating the extension and closure of the isolation membrane and further fusion of autophagosomes with lysosomes. These proteins are essential for starvation-induced autophagy as well as selective autophagy. In the following section, we will summarize the molecular details of autophagy, which are required to understand the underlying mechanisms of each hereditary disorder associated with mutations in autophagy-related genes.

**Table 1:** Homologs of autophagy-related genes.

	*S. cerevisiae*	Mammals					
ULK1 complex	Atg1	ULK1	ULK2	ULK3	ULK4		
	Atg13	ATG13					
	Atg17	FIP200					
	–	ATG101					
PI3K complex	VPS34	VPS34					
	VPS15	VPS15					
	Atg6	Beclin 1					
	Atg14	ATG14	UVRAG				
	–	Ambra1					
	–	Rubicon					
PI3P-binding proteins	Atg18	WIPI1	WIPI2	WIPI3 (WDR45B)	WIPI4 (WDR45)		
ATG9 vesicles	Atg9	ATG9L1	ATG9L2				
LC3 conjugation system	Atg8	LC3A	LC3B	LC3C	GABARAP	GABARAPL1	GABARAPL2/GATE-16
	Atg4	ATG4A	ATG4B	ATG4C	ATG4D		
	Atg3	ATG3					
	Atg7	ATG7					
	Atg10	ATG10					
	Atg5	ATG5					
	Atg12	ATG12					
Others	Atg2	ATG2A	ATG2B				

### Induction of autophagy

Nutrient starvation is the best-characterized stressor inducing autophagy. The release from negative regulation by the mTOR complex triggers autophagic induction. Under normal nutrient conditions, ULK1 complex is inactivated due to its constitutive phosphorylation by the mTOR complex. The high amino acid concentration in the lysosome keeps the mTOR complex activated in a manner dependent on lysosomal proton pump v-ATPase complex, which promotes the binding of Rag GTPase with mTORC1. When cells are exposed to starvation conditions, decreased amino acid levels in the lysosomes makes mTORC1 to be released from the lysosomes, resulting in reduced phosphorylation of its targets^5,6^. Concomitantly, de-phosphorylation of ULK1 by phosphatase PP2A promotes activation of ULK1 complex^7,8^. In yeast, the phosphorylation changes the complex formation as phosphorylation of Atg13 by TOR inhibits the binding of Atg1 with Atg13^9^. In mammalian cells, the phosphorylation does not halt the formation of the ULK1-ATG13-FIP200 complex but instead inhibits the kinase activity of ULK1 complex via regulating the binding of ULK1 complex with AMPK^7,10^. mTORC1 also regulates autophagy through transcription factors. Under normal nutrient conditions, the transcription factor TFEB is phosphorylated by mTORC1 that facilitates its localization in the cytoplasm through binding to the 14-3-3 protein. When this phosphorylation decreases during starvation, TFEB is transferred to the nucleus, which can induce the transcription of autophagy- and lysosome-related genes^11,12^. In addition to TFEB, another transcription factor, TFE3, also translocates to the nucleus in response to starvation to activate autophagy^13^. In summary, the mTOR pathway plays a central role in the control of autophagy. However, some pathways regulate autophagy independent of mTOR; and thus, the mechanism of autophagic activation is not always the same and varies in response to different kinds of cues^14^.

### Origin of the isolation membrane

The source of autophagosomes has been a hot topic in the field of autophagy. The best-known source is the endoplasmic reticulum (ER). Autophagosomes have been proposed to be formed by extending the isolation membrane along with the ER, which is called the “cradle model”. This hypothesis is supported the three-dimensional structure generated by electron tomography^15^. It is generated from an extended ER membrane in a form of Ω-shaped structure called omegasome^16^. The shaping of omegasome and autophagosomes is supported by an assembly of actin inside these structures^17^. Consistent with the fact that mitochondria can also be a source, autophagosomes are formed at the contact sites between the ER and the mitochondria, as shown by simultaneous imaging of three organelle markers^18,19^. Other materials, such as cell membranes and lipid droplets, are also considered sources for autophagosomes^20–22^. ATG9 vesicles and the ER-Golgi intermediate compartment (ERGIC) are believed to be involved in supplying materials for isolation membranes^23–26^. To facilitate the source supply and the shaping of autophagosome, two upstream components play a major role: ULK1 complex and ATG9 recruit downstream factors and coordinate the events as described below.

### Upstream factors of autophagy: membrane sources and formation of isolation membranes

In yeast, the pre-autophagosomal structure (PAS) is formed at the proximal area of the vacuole, where ATG proteins accumulate to generate isolation membranes^27^. The mechanism of their action was investigated by intracellular localization analysis of Atg proteins with fluorescent tags in the absence of each gene^28^. Based on this model, the hierarchy of the molecular actions of mammalian autophagy-related proteins was established. Mammalian cells do not form a clear PAS like yeast cells, but each autophagy-related protein accumulates at distinct sites where the isolation membrane is generated. These sites are observed by the formation of dots under fluorescence microscopy. As described above, phosphorylation of ULK1 complex is the most upstream target for autophagic induction and is located upstream of the cascade^29^. ATG9 is the only membrane protein in the core ATG proteins and is contained in a small membrane structure of ~30–60 nm called ATG9 vesicles. To initiate autophagosome nucleation, both ULK1 and ATG9 are localized at autophagosome formation sites that are supposed to be contact sites of ER and other organelles such as mitochondria^19,30^. Under the nutrient-rich conditions, ATG9 travels through plasma membranes, early endosomes, recycling endosomes and trans-Golgi network (TGN). Upon induction, a portion of it localizes to autophagosome formation site from recycling endosomes or TGN. It depends on TRAPPIII complex that mediates retrograde trafficking from endosomes to Golgi^31–33^. Although ATG9 vesicle itself does not become a membrane source, it facilitates the delivery of membrane sources for autophagosomes^23^. A recent study using a proteomics approach identified contents of ATG9 vesicles, showing that it is enriched in BAR-domain-containing proteins including Arfaptin 2^34^. It supports the distribution of ATG9 vesicles and the delivery of PI4K and PI4KIIIβ to autophagosome formation sites.

Given that knockdown of ATG9 results in only a slight reduction in ULK1 dot formation^23^, and that ATG9 and ULK1 complex are independently targeted to depolarized mitochondria for Parkin-dependent mitophagy^35^, ULK1 and ATG9 are not essential for their initial recruitment to the site of autophagosome formation each other. However, ULK1 complex is needed for the trafficking of ATG9 vesicles as shown by the retention of ATG9 on TGN in the absence of ULK1 even in starved conditions^36^. It is mediated by phosphorylation of ATG9 on Ser14 by ULK1 upon starvation signal as is the case with yeast showing that Atg1 phosphorylates Atg9 to facilitate recruitment of downstream components to PAS^37,38^. Interestingly, knockdown of PI4KIIIβ leads to a reduction in dot formation of ATG13, a component of ULK1 complex, in starved condition, suggesting that ATG9 supports recruitment of ULK1 complex by promoting PI4P enrichment on the site of autophagosome formation^34^. Thus, it is most likely that those two major factors are required for their full activity to facilitate autophagy. It is not only at the step of initiation of isolation membrane formation but also elongation of the membrane. It has been known that ATG2A, which is required for supplying lipid to autophagosome membrane to facilitate the elongation^37–40^, works to establish ATG9 localization on mitochondria-associated ER membranes^41,42^. It is also the case of ULK1 complex because this complex phosphorylates a wide variety of substrates related to autophagosome biogenesis that are involved in the initiation step to the fusion step as well^43^.

Recently, it has been clear that PAS is a liquid-like condensate of Atg proteins. Upon starvation signal, the de-phosphorylation of Atg13 results in an establishment of Atg1 complex and formation of a liquid droplet of early PAS. This phase separation ensures Atg1 kinase activation and subsequent recruitment of other Atg proteins to make matured PAS^44^. As ATG9 is embedded in ATG9 vesicles that only transiently localize to the vicinity of autophagosome^23,45^, one can speculate that ATG9 vesicles exist as a distinct component from the liquid droplet, and ATG9 on the membrane might temporally contact with ATG2A, ULK1 or other autophagy-related proteins in the liquid droplet. Thus, it is important to understand a topological basis which ensures ATG9 vesicles to access to other ATG proteins in the liquid droplet and promote a range of events described above.

Taken together, coordinated actions of ATG9 and ULK1 complex are inevitable for autophagy through regulating membrane source supply and recruitment of downstream components, promoting proper autophagosome maturation.

### Production of PI3P by the PI3K complex and extension of the isolation membrane

Phosphatidylinositol 3-phosphate (PI3P), generated by PI3K, is a phospholipid that plays an important role in the control of membrane trafficking. While yeast has only one PI3K (Vps34), mammalian cells have several PI3Ks, of which class III PI3K is involved in autophagy. This is composed of the complex containing ATG14 or UVRAG in addition to VPS34, VPS15, and Beclin 1 (VPS30 homolog)^46–48^. While the PI3K with UVRAG is involved in the autolysosome formation, that with ATG14 promotes both formation of isolation membrane and the later steps as well^47–49^. PI3P promotes the localization of proteins with PI3P-binding motifs such as FYVE domains to the membrane where PI3P is embedded. Isolation membranes require the recruitment of four ATG18 homologs, WIPI1-4 that interact with PI3P through FRRG motif for WIPI1 and WIPI2 or LRRG motif for WIPI3 and WIPI4^43^. Since these paralogs may have not only a functionally redundant role as shown by localization of all of four paralogs on nascent autophagosomes upon starvation, but also specific roles as well, since phenotypes caused by the loss of each gene could not be fully compensated by the presence of the other paralogs. WIPI2 acts as a scaffold to recruit ATG16 to isolation membrane, WIPI3 binds to TSC complex and FIP200, and WIPI4 interacts with ATG2A to facilitate autophagosome formation^37,39,40,50,51^. Consistent with this idea, mutations in the *WIPI4* (*WDR45*) gene cause static encephalopathy of childhood with neurodegeneration in adulthood (SENDA), a human genetic disease involving neurodegeneration^52^. *WIPI2* gene is also known to be responsible for a hereditary disease showing neurological disorders (described below)^53^. FYVE domain-containing protein DFCP1 is often used as a marker of isolation membranes but is not functionally involved in autophagy itself^16,54^. The function of PI3K in the formation of the isolation membrane depends on ULK1 and ATG9 since the formation of ATG14, DFCP1 or WIPI2 dots decrease with deficiencies in the components of ULK1 complex or ATG9^23,29^. By these actions, omegasome is formed from the ER, from which a bag-shaped isolation membrane is developed^16^.

### Post-translational modification of LC3

The regulation of autophagosome formation and the subsequent events are governed by a characteristic reaction involving the post-translational modification of the Atg8 homolog LC3 with phosphatidylethanolamine (PE). It is mediated by a series of ubiquitin-like modification enzymes^55–57^(reviewed by N. Mizushima in detail^58^). This reaction is carried out via two pathways. The first pathway is a modification of the ubiquitin-like protein ATG12, which is covalently bound to ATG5 via the action of the E1-like enzyme ATG7 and the E2-like enzyme ATG10. Most ATG12 proteins exist in a form bound to ATG5. The ATG5-12 complex binds to ATG16. The ATG5-ATG12-ATG16 complex functions as an E3-like enzyme in the last step of another ubiquitin-like reaction to facilitate modification of LC3 with PE. The ubiquitin-like protein LC3, a homolog of the budding yeast Atg8, is first cleaved by the ATG4 protein at the C-terminus to expose the Gly residue. Through sequential reactions of ATG7 (E1), ATG3 (E2), and the ATG5-ATG12-ATG16 complex (E3), PE is added to the C-terminal glycine residue of LC3 to target it to the isolation membrane.

Membrane-bound LC3 with PE functions as a key molecule that controls critical steps in the molecular mechanism of autophagy: autophagosome closure^59,60^, movement of the autophagosomes^61–64^, fusion with the lysosomes and degradation of the inner membrane of the autophagosomes^65–68^. This molecule is also needed for the binding to adapter proteins in selective autophagy. LC3 homologs are mainly classified into the LC3 subfamily and GABARAP subfamily. Knockdown of the Atg8 homologs of each subfamily with siRNA reduced autophagic activity, which suggests some functional segregation^69^. Moreover, it is recently shown that LC3 subfamily and GABARAP subfamily have opposite roles for regulation of autophagosome formation via binding to ULK1 and ATG13^70^.

While other ATG proteins are located outside the isolation membrane and move away from the membrane after autophagosome formation, LC3 is located both outside and inside the isolation membrane and stays within the autophagosome. Therefore, LC3 itself is degraded by autophagy, making it a useful substrate to measure autophagic activity. As LC3-I (without PE) and LC3-II (LC3-PE) can be easily separated from each other by SDS-PAGE, an autophagic flux assay using LC3 is widely used as a standard method to measure autophagic activity^71^. LC3 is the best-known marker used to monitor the formation of autophagosomes by microscopic observation as well^72^. The use of fluorescent-tagged LC3 enables the observation of autophagosomes in living cells. Specifically, a tandem-tagged LC3 with both GFP and RFP has been developed to distinguish autophagosomes from autolysosomes, which is critical to determine the state of autophagic induction or inhibition. In the case of lysosomal inhibition, LC3 dots accumulate, which cannot be distinguished from elevated levels of LC3 due to autophagic induction. Because the GFP signal is quenched in lysosomes due to its pH-sensitive nature, RFP-GFP-LC3 shows green plus red for autophagosomes and red with no green signal for autolysosomes^73^ (the plasmid is available from Addgene, plasmid # 21074). Thus, a series of methods using LC3 as a marker for autophagosomes has substantially advanced research on mammalian autophagy^71^.

### Fusion with lysosomes

Not only autophagosome formation but also the subsequent fusion with lysosomes is controlled in a highly orchestrated manner. The fusion event requires physical contact of both organelles, and autophagosomes dynamically travel throughout the cytosol because lysosomes are predominantly generated at a peripheral region of the nuclei, while autophagosome formation sites are not restricted to the area where lysosomes are present. Inhibition of LC3 by injection of an LC3 antibody prevented the movement of autophagosome, suggesting that LC3 is needed for this event^61^. It is mediated by a binding of LC3 with FYVE and coiled-coil domain containing protein (FYCO1), which tethers autophagosomes with kinesin and microtubules to facilitate the plus-end direction of movement (peripheral direction)^64^. The movement of autophagosomes in the perinuclear direction is mediated by the binding of autophagosomes with dynactin and dynein via Rab7, ORP1L, and RILP^62,63^. Interestingly, a lack of ORP1L caused decreased recruitment of the components required for the following fusion event described below to late endosomes or lysosomes^63^, suggesting that the movement and the fusion are not separated from each other but are instead regulated as a sequential event. To date, researchers have shown that SNARE complexes containing Stx17 and the HOPS complex are required for the tethering of autophagosomes with lysosomes to facilitate the formation of autolysosomes^74^. This process is also under the control of the abovementioned PI3P production by PI3K. Beclin 1/VPS34/VPS15 with UVRAG functions as a mammalian PI3K complex working at the fusion step. Interestingly, PI3K with UVRAG is controlled by binding to a protein called Rubicon^48,75^. Rubicon knock-down results in an upregulated autophagic flux and a promotion of the fusion process, suggesting that Rubicon is a negative regulator of the fusion step. Rubicon itself is regulated by other factors, including mTOR, indicating that it is a key regulatory factor of autophagy whose deregulation may cause deleterious effects in pathology^76–78^. Indeed, Rubicon protein showed excessive accumulation in fatty liver induced by a high-fat diet, and a genetic abrogation of Rubicon suppressed diet-induced liver diseases, suggesting that abnormal accumulation of Rubicon may be a cause of these diseases^79^. The tethering and fusion of autophagosomes and lysosomes are mediated by two regulatory factors, EPG5 and INPP5E, which are known to be responsible for two human hereditary disorders, Vici syndrome and Joubert syndrome, respectively. EPG5 is present in late endosomes and lysosomes, facilitating the fusion of both by promoting the assembly of the SNARE complex, which is required for autophagosome-lysosome fusion^80^. INPP5E is a phosphatase that converts PI(3, 5)P2 to PI3P. The downregulation of INPP5E resulted in a reduction in the autophagic flux associated with an accumulation of LC3 dots and reduced colocalization of LC3 with lysosomes, showing that INPP5E facilitates autophagy via regulation of autophagosome-lysosome fusion^81,82^. Consistent with the fact that actin polymerization facilitates autophagy^17^, PI(3,5)P2 regulates the endosomal actin dynamics by antagonizing cortactin binding to actin filaments^83^, and reduction in cortactin decreased autophagosome-lysosome fusion^84^, downregulation of INPP5E resulted in reduced activation of cortactin on lysosomes. This evidence suggests that the conversion of PI(3,5)P2 to PI3P by INPP5E on lysosomes may increase the stability of actin filaments in a cortactin-dependent manner, promoting the fusion of autophagosomes with lysosomes.

### A physiological role for autophagy in humans; lessons from animal models and human hereditary disorders

The physiological significance of autophagy has been investigated using various model animals, including mice, zebrafish, fruit flies and nematodes. In the case of mice, these studies are mainly performed using models in which the genes essential for autophagy are specifically knocked out for each tissue because knockout mice with a systemic homozygous deletion in these genes show embryonically lethal or postnatal lethality^85^. To date, over 150 types of tissue-specific autophagy-related gene knockout mouse models have been generated, suggesting the important roles of these genes in maintaining homeostasis in each tissue. Moreover, studies on mice with an *Atg5* homozygous deletion in a mosaic throughout the body^86^, tamoxifen-induced knockout mice after birth^87^, and mice that express *Atg5* only in the nervous system^88^ suggest a comprehensive role for autophagy throughout the body. These findings emphasized that autophagy ensures the normal development of tissues and the maintenance of homeostasis of differentiated tissues, and the failure of this process leads to dysfunction of each organ in mammals.

What about its role in human beings? Direct evidence has been provided by human hereditary diseases. These diseases are caused by hypomorphic mutations in the genes essential for autophagy or loss-of-function mutations in genes related to but not essential for autophagy. No genetic disease linked to a lack of both alleles of genes essential for autophagy has been reported to date. This phenomenon is most likely because autophagic deficiency affects the survival of human individuals, as shown in the results of embryonic lethality or postnatal lethality in mice with systemic homozygous deletions in these genes. Here, we will summarize the features of each disease and discuss the physiological role of autophagy in human health.

### SENDA

**Fig. 2:**
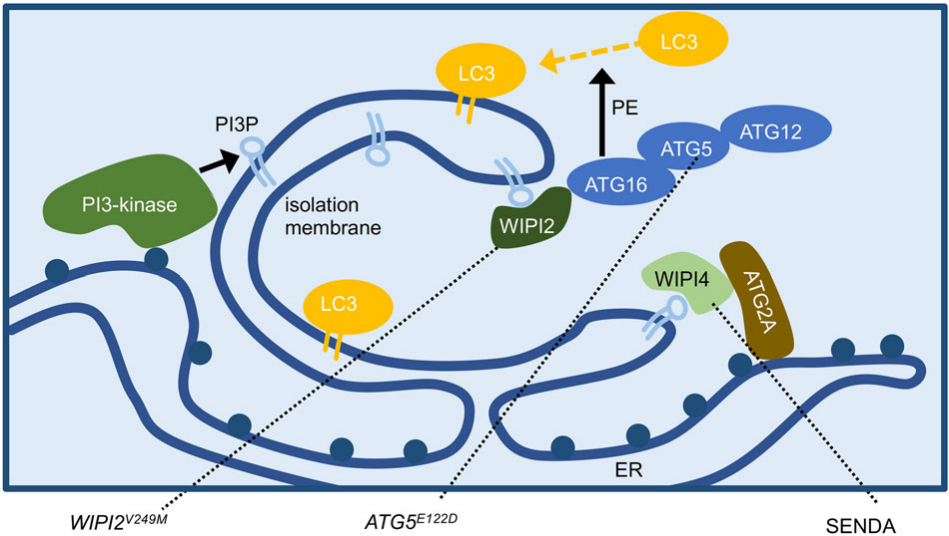
Multiple proteins involved in autophagosome biogenesis are responsible for the suppression of human hereditary disorders involving neurodegeneration. The formation of the isolation membrane is initiated at the contact sites of the mitochondria and the ER, followed by elongation of the membrane in a structure called the omegasome. PI3P production by PI3K facilitates the recruitment of WIPI2 that further promotes localization of ATG16 at isolation membranes for subsequent modification of LC3. WIPI4 also binds to PI3P to facilitate autophagosome biogenesis via binding to ATG2A, supporting the retention of the omegasome with the ER^40^ or a feeding of lipid to isolation membrane^39^.

Neurodegeneration with brain iron accumulation (NBIA) is a rare degenerative disease (~1/1,000,000) characterized by iron deposition in the brain, and SENDA is one of its subtypes. Psychomotor development is delayed early in childhood. Motor function develops to some extent, but dystonia, parkinsonism, and dementia progress rapidly in adolescence or early adulthood^89^. Most SENDA is sporadic, and thus, de novo mutations are expected to be responsible for its symptoms. Saitsu et al. conducted an exome analysis of two SENDA families and identified mutations in *WDR45* (*WIPI4*), a human homolog of *Atg18*, one of the core genes essential for yeast autophagy^52^ (Fig. 2). The WDR45 locus is located on the short arm of the X chromosome (Xp11.23). Although X chromosome inactivation should be heterogeneous in each cell, lymphoblastoid cell lines (LCLs) from 4 of the 5 patients showed expression of only the mutant form. Thus, this factor is believed to be related to the disease state, but the mechanism is still unclear. There are four *Atg18* homologs, WIPI 1 to 4, in humans. The phenotype of the *WDR45* neuron-specific knockout mice was slightly milder than that of the mice with knockout of the core genes such as *ATG5* and *ATG7*, each of which has only one ortholog, suggesting some functional overlap among the paralogs^90^. However, while WIPI2 binds to ATG16, which is required for the PE reaction of LC3, WIPI4 binds ATG2A more strongly than the other WIPI proteins, showing some functional differences^50,91^. In a mutant of *epg-6* (a nematode homolog of *WIPI4*), the accumulation of protein aggregates and the multi-layered membrane-like structures were observed, suggesting the role of WIPI4 in autophagosome formation in vivo^92^. A recent study showed that *Wdr45* knockout mice have increased neuronal apoptosis due to an accumulation of ER proteins, which was associated with an increase in the unfolded protein response (UPR)^93^. An investigation of the link between ER stress and autophagy indicated that ER stress can be one of the cues that induces autophagy^94^.

### Developmental abnormalities associated with the homozygous *WIPI2*^*V249M*^ mutation

Since a loss of *WIPI2* results in a defect in LC3 lipidation and the associated autophagosome formation, WIPI2 may have a distinct function in autophagy among human Atg18 paralogs^54^. Recently, individuals with a homozygous mutation in *WIPI2* were reported to show developmental abnormalities and mental retardation^53^. Exome sequencing of two affected individuals identified the G745A mutation in exon 9 of the *WIPI2* gene, which leads to a V249M amino acid change in the gene product. This mutation causes reduced binding activity of WIPI2 with ATG16L1, which is known to facilitate recruitment of ATG16L1 to the site of autophagosome formation and thus promote LC3 lipidation and autophagosome biogenesis^50^. The affected individuals analyzed for exome sequencing were aged 43 years and may have residual autophagosome biogenesis activity, as fibroblasts from the patient had mild but still induced LC3 lipidation activity in response to a starvation signal. The individuals with the mutation showed a complex developmental disorder including mental retardation, speech and language impairment, and cardiac, neurological, thyroid and skeletal abnormalities. Characteristic features of the disease are severe mental retardation associated with global brain volume loss and short stature. Cerebellar dysfunction is suggested, as fine/gross motor activities are delayed in patients. Cardiac abnormalities shown by electrocardiograms were observed^53^. The clinical features were similar at some degree in the patients with the *WIPI2* mutation and those with SENDA although the patient with the *WIPI2* mutation did not show iron deposition, a characteristic feature of SENDA.

### Congenital ataxia with a homozygous *ATG5*^*E122D*^ mutation

Mutations in the genes essential for autophagy among the core autophagy-related genes have not been shown to be related to human diseases until the identification of the *ATG5*^*E122D*^ mutation. Exome analysis of two families with congenital ataxia (ataxia), intellectual disability, and growth retardation identified the chr6: 106,727,648 T > A mutation, a missense mutation that causes an E122D change in the ATG5 protein^95^. ATG5 is covalently bound to ATG12 to form a heterodimer of a ternary complex with ATG16, which promotes lipidation of LC3^55,58^. Since the *ATG5*^*E122D*^ mutation weakens the binding to ATG12 and decreases PE formation of LC3, basal autophagic activity was reduced in the LCLs derived from patients, and accumulation of p62 was observed^95^. These data showed that *ATG5*^*E122D*^ is a hypomorphic allele whose homozygous mutation results in reduced but residual autophagic activity. The reduction in autophagy is most likely sufficient to support survival after birth but insufficient to maintain homeostasis in the CNS and suppress the symptoms of this condition. In contrast to SENDA, this disease did not show significant cerebral atrophy. Intriguingly, while cerebellar atrophy is a characteristic feature of the patients *ATG5*^*E122D*^, it is not a major feature of SENDA as nearly one third of the patients of SENDA show the symptom^89^. This controversy might be explained by the tissue-specific function of the *WDR45* gene or genetic diversity (see below)^96^. No iron deposition in the cerebral area of the patient was reported.

### Vici syndrome

Vici syndrome is an autosomal recessive disorder caused by mutations in the *EPG*5 gene, which has a regulatory role in autophagy at the fusion step between autophagosomes and lysosomes^97^. This condition affects not only the corpus callosum but also a wide range of organs and can include cardiomyopathy, cataracts, depigmentation, and combined immunodeficiency. Ectopic P-Granules Autophagy Protein 5 (EPG5) is a metazoan protein identified by screening *Caenorhabditis elegans*, and its homolog has not been found in yeast^98^. Systemic knockout mice that lack both alleles of *Epg5* are born according to Mendelian inheritance and survive after birth^99^. This phenotype is clearly distinguished from those caused by the core autophagy genes, such as *Atg5* and *Atg7*, which include neonatal lethality due to defects in neurological activity related to sacking defects and survival with starvation after birth^88,100,101^. This difference is probably due to the weak remaining autophagic activity that mitigates the phenotype in the *Epg5* knockout mice^99^. The knockout mice began to show motor dysfunction after approximately 4 months and then showed symptoms similar to amyotrophic lateral sclerosis (ALS), such as muscle fiber atrophy and progressive paralysis^99^. Notably, the *Epg5*^*−/−*^ mice showed only some features that are observed in patients with Vici syndrome. The mice showed agenesis of the corpus callosum and muscle atrophy but lacked several key symptoms of Vici syndrome, including facial dysmorphism, cataracts, and hypopigmentation. Additionally, *Epg5* knockout mice can grow and reach sexual maturity in contrast to those with Vici syndrome, in whom death occurs at a median age of 42 months^98,102^. This observation that abrogation of *EPG5* causes a less severe phenotype in mice than human support a speculative idea that human might depends on autophagy more than mice to maintain tissue homeostasis.

**Fig. 3:**
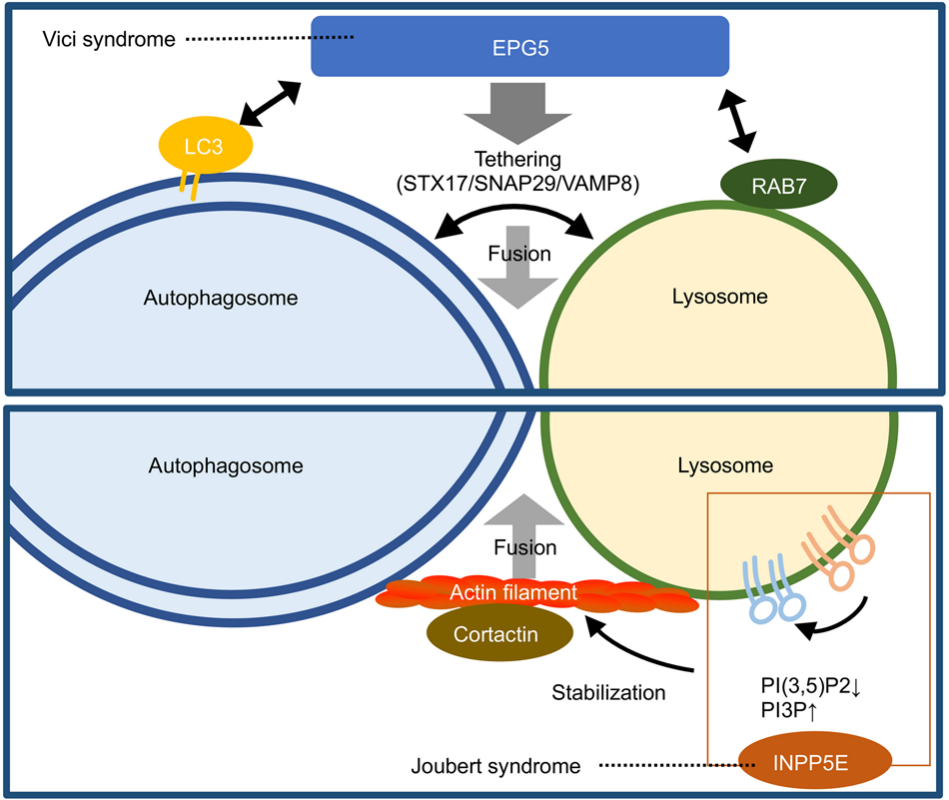
Two regulators of autohagosome–lysosome fusion, EPG5 and INPP5E, are responsible for human hereditary disorders. *EPG5*, which is responsible for Vici syndrome, is required for the tethering and fusion of autophagosomes and lysosomes. EPG5 facilitates the tethering by direct binding to and assembly of the SNARE complexes composed of STX17, SNAP29 and VAMP8. Jourbert syndrome patients have a mutation the in *INPP5E* gene. Its gene product INPP5E, a phosphatase, catalyzes the conversion of PI3P to PI(3, 5)P2 on lysosomes, resulting in of the binding of cortactin to actin filaments to stabilize this structure.

EPG5 promotes tethering of autophagosome and lysosome^80^ (Fig. 3). In *EPG5* knockout cells, autophagosomes fuse with early endosomes, not lysosomes or late endosomes, and an enlarged autophagosome-like structure accumulates. Similar structural accumulation is observed in *Epg5* knockout mice and tissues from patients with Vici syndrome. In addition, the accumulation of p62 and ubiquitin-positive dots was observed. Although further analysis is needed, the formation of these autophagosome-like structures and protein aggregates may contribute to the pathology of Vici syndrome.

### Joubert syndrome

Joubert syndrome is characterized by abnormal eye movements, decreased muscle tone, and delayed psychomotor development and brainstem dysplasia called the molar tooth sign. In addition to neuropathy, a wide range of symptoms, such as kidney damage and liver damage, are observed^103^. Bielas S et al. found that patients belonging to multiple families shown to have Joubert syndrome locus 1 (JBTS1) loci have mutations in the *INPP5E* gene^104^. INPP5E is an enzyme that generates PI3P by dephosphorylating the 5-position of PI (3,5)P2, which is a kind of inositol phospholipid (PIP). As described above, PI3P plays an important role in autophagosome formation and autophagosome maturation. Screening for factors related to autophagy among the factors involved in the metabolism of PIPs showed that INPP5E was required for autophagosome maturation^81,82^ (Fig. 3). Cells with mutations similar to the INPP5E mutation found in patients showed a significant decrease in autophagic activity, strongly suggesting that autophagic abnormalities may cause Joubert syndrome^82^. Cells from patients with Joubert syndrome showed abnormalities in the maintenance of cilia; thus, Joubert syndrome is believed to be associated with ciliopathy. Notably, autophagy was reported to be involved in the regulation of primary cilia formation and length, and conversely, signaling from cilia regulates autophagy^105^; thus, feedback of autophagy and cilia abnormalities may have affect the expression of the symptoms.

### The physiological role of autophagy in homeostasis in humans

Although tissue-specific knockout of autophagy-related genes in mice has provided a clear-cut role for autophagy in tissue homeostasis, to develop clinical applications of autophagy induction or inhibition for related diseases, the physiological/pathological consequences of systemic changes in autophagy must be elucidated. In addition, recent studies have shown that autophagy declines with age. Knowledge of autophagy-associated human genetic diseases will help elucidate this issue. Consistent with the idea that systemic, amorphic mutations of genes essential for autophagy may result in lethality, patients with autophagy-related mutations retain residual autophagic activity, suggesting that the symptoms shown above are caused by partial decline in autophagic activity. Therefore not showing clear-cut mechanisms as shown in the tissue-specific knockout mice, those results provide physiologically relevant information. Based on these findings, we discuss the function of autophagy in human health, especially brain function, development, and tumorigenesis.

### Brain function and development

The most striking shared feature in the disorders shown above is an aberration in the CNS. This finding is consistent with a role for autophagy in the prevention of neurodegeneration shown by mice with neuron-specific homozygous deletions in *Atg5*^106^. These mice showed accumulation of protein aggregates in the neurons, neuronal loss, motor dysfunction, and neurodegenerative disease symptoms. In the case of a systemic loss of autophagy in mice by inducible deletion of *Atg7* after birth using the inducible Cre-lox system, abnormalities were observed in many tissues, such as the liver, brain, muscles, and pancreas^87^. However, except for some deaths caused by infection, most mice died ~3 months after the induction of deficiencies in autophagy due to neurodegeneration, supporting the idea that autophagy is critical for the CNS^87^. This phenomenon is not limited to mammals, as *Drosophila Atg5* or *Atg7* mutants also exhibited neurodegenerative disease-like symptoms, highlighting the general role of autophagy in maintaining neuronal homeostasis^95,107^.

The reason why autophagic deficiency primarily disrupts the CNS is unclear, but it may be due to two specific characteristics of the CNS. First, a defect in the clearance of cellular wastes may cause a strong deleterious effect in neurons, which rarely divide throughout life and thus are exposed to a cumulative toxic effect of the debris. In particular, the cells in the CNS may be strongly affected in long-lived humans compared to mice. Another reason may be the highly specified function of each cell in the CNS, which has less redundancy than other organs, such as the liver.

While the congenital diseases described above have some shared characteristics, there are significant differences in the severity and types of symptoms. Among them, Vici syndrome displays the most severe condition, followed by Joubert syndrome and SENDA. The ataxia associated with *ATG5*^*E122D*^ and the developmental abnormalities associated with the homozygous *WIPI2*^*V249M*^ mutation are relatively mild. Although still speculative due to a lack of a cross-cutting study involving the diseases shown here, the degree of severity may depend on the levels of residual autophagic activity in the CNS. Indeed, fibroblasts from patients with Vici syndrome showed almost no induction of autophagic flux by rapamycin and a strong accumulation of the autophagic substrate p62 and NBR1 in normal conditions^97^. LCLs from patients with the *ATG5*^*E122D*^ mutation showed a decrease in LC3 lipidation and autophagic flux but still retained a detectable level of flux^95^. This finding was also observed for those with the *WIPI2*^*V249M*^ mutation^53^. Among INPP5E knockout cell lines complemented by the transgenes with some mutations found in patients with Joubert syndrome, some of mild symptoms showed a slight but significant recovery in autophagic activity, but the severe symptoms did not show any recovery, showing a correlation between the enzymatic activity and the severity of symptoms^82^.

The characteristics of symptoms in the CNS also vary. SENDA is characterized by iron deposition in the CNS, which is not found in the other four diseases. While agenesis of the corpus callosum is typically found, and atrophy of cerebrum is not a major feature of the patients with Vici syndrome, patients with the *ATG5*^*E122D*^ mutation show cerebellar hypoplasia without agenesis of the corpus. SENDA also shows cerebellar atrophy. This controversy may be explained by several reasons: (1) gene-specific roles other than autophagy, (2) genetic diversity and (3) tissue specificity of each gene.

Regarding (1) the gene-specific roles other than autophagy, the Vici syndrome-related protein EPG5 has a role in the regulation of endocytosis, which has a critical function in neural development and neurons. Similarly, the Joubert syndrome-associated protein INPP5E catalyzes the synthesis of PI3P from PI(3,5)P2, as PI(3,5)P2 and PI3P have physiological functions other than autophagy, including the regulation of endosomal function^108^. Deletion of FIG4, which facilitates the production of PI(3,5)P2, resulted in a depletion of PI(3,5)P2 and developmental defects including neurological abnormalities. Its homozygous mutation has been identified as a cause of Yunis-Varon syndrome, which is characterized by structural brain abnormalities and skeletal abnormalities^109^. Mutation of *FIG4* was also found in mutant mice called “tremor mice” that show a severe tremor and abnormal gait, as well as in patients with the hereditary condition Charcot-Marie-Tooth disorder^110^. Therefore, it is likely that a defect in endocytosis may cause an additive effect on brain development, resulting in the complex symptoms found in Vici syndrome and Joubert syndrome. This phenomenon may also be the cause of symptoms in organs other than the brain, such as the immunological abnormalities in Vici syndrome and the liver disease in Joubert syndrome that are not found in SENDA or patients with *ATG5*^*E122D*^ mutations. Notably, ATG5 also has roles in processes other than autophagy, such as cell death and pathogen resistance^111,112^, which could contribute to the symptoms of patients with the *ATG5*^*E122D*^ mutation to some degree.

Regarding the #2 possibility, genetic diversity including quantitative trait loci (QTLs) is expected to contribute to variations in symptoms because autophagy is a multistep event requiring several different kinds of biological pathways. Membrane trafficking, including endocytosis, is a major contributor to autophagy because it regulates the maturation of autophagosomes. Genes involved in mitochondrial biogenesis, lysosomal biogenesis, and hormonal regulation are required for the proper regulation of autophagy. For instance, MFN2, a mitochondrial dynamin-related protein that is required for the tethering of the mitochondria with the ER^113^ and the formation of isolation membranes^19^, is known to be mutated in Charcot-Marie-Tooth neuropathy type 2A^114^. Multiple genetic variations in this wide range of factors may contribute to the differences in severity and tissue specificity. We expect that genetic variation in other genes may be related to the symptoms but independent of autophagy. For example, since genetic loci of *BECN1* (encoding beclin 1) is close to a well-known tumor suppressor *BRCA1*, large deletions including both loci are found in the cancer genome^115^.

For the #3 possibility, the tissue specificity of each gene may also contribute to the symptoms. Mice with double systemic homozygous deletions in both *Wdr45* and *Wdr45b* showed neonatal lethality, while each single knockout mouse was born normally, suggesting functional overlap between the genes^96^. Double knockout mice showed an increased accumulation of p62 aggregates in many brain regions and neurons, which was associated with suckling defects similar to those of *Atg7* knockout mice^100^. Interestingly, these accumulations of the p62 aggregates were not detected in the liver and kidney in double knockout mice, suggesting that both genes may not be essential for autophagy in these tissues^96^. Therefore, the functional contribution of each autophagy-related gene may vary in different tissues.

In summary, the shared feature of dysfunction in the CNS in various diseases highlights the critical role for autophagy in the maintenance of homeostasis in neurons for CNS function. However, the possibilities shown above (which are not mutually exclusive) suggest that some abnormalities found in Vici syndrome, such as cataracts and severe immunological abnormalities, and Joubert syndrome, such as renal disease and liver disease, might be due to combined defects in autophagy and endocytosis, as well as other unidentified genetic factors(s). A comprehensive study using humanized mouse models for each disease with a defined genetic background may help elucidate the role of autophagy in the suppression of these diseases and human physiology as well.

### Tumorigenesis

Several studies of tissue-specific knockout mice have identified a tumor-suppressive role for autophagy in many tissues, including the liver, lung, and pancreas^86,116,117^. Due to the prosurvival effect of autophagy on tumor cells, tumors arising from these mice usually do not develop into malignant tumors. However, these findings do not necessarily indicate that autophagy is dispensable for tumor suppression. Rather, an irreversible genetic abrogation of the genes essential for autophagy in these model systems clearly show both side of autophagy in tumorigenesis^118^. Nonetheless, the human genetic disorders with mutations in autophagy-related genes described here do not show a significant increase in any sign of neoplasm formation. Do these findings indicate that autophagy may not be important for tumor suppression in humans? Evidence from cell biology using human cells clearly shows that autophagy is required to suppress the genomic alterations that promote tumorigenesis. Autophagy promotes genomic integrity through the control of cellular senescence via both positive and negative regulation. Promoting cellular senescence in response to oncogenic stress^119^, autophagy also suppresses senescence by selective degradation of the transcription factor GATA4 in response to DNA damage^120^. Autophagy also maintains stemness of muscle stem cells by suppression of senescence caused by excess ROS production^121^. Alternatively, autophagy triggers cell death in response to telomeric DNA damage during replicative crisis^122^. These lines of evidence suggest that autophagy suppresses tumor-promoting genomic alterations in various processes. Therefore, we hypothesize that the controversy between human symptoms and the phenotypes of mice with tissue-specific, irreversible deletions in autophagy genes is most likely due to a major contribution of autophagy to the maintenance of the CNS in humans, which is vital for survival. Simply, a reduction in only a fraction of the CNS may immediately cause a serious defect, in contrast to carcinogenesis, which requires a long period to acquire multiple mutations for the development of a malignant tumor. Indeed, a mouse model with temporal systemic inhibition of autophagy followed by its restoration supports this idea. The mice with reduced expression of *Atg5* by dox-inducible siRNA after birth expressed reduced viability (~6 months) and various signs of accelerated aging due to systemic degradation of tissue homeostasis. Interestingly, the restoration of autophagy in mice partially restored the shortened lifespan but accelerated the formation of spontaneous tumors^123^. We speculate that a systemic decrease in autophagy becomes a serious threat to survival, especially due to a defect in the CNS that precedes the formation of neoplasms, but a temporal but severe decrease in the autophagy of cells in a limited area, such as the damaged liver, may cause genomic alterations that facilitate tumorigenesis.

A complex tumor environment and the heterogeneity of cancer cells must be considered for the role of autophagy in tumorigenesis. Autophagy not only suppresses genomic instability or conversely supports cancer cell survival in a cell-autonomous manner but also controls tumor cell status in a non-cell autonomous manner. Autophagic activity in host mice is required for the maintenance of circulating arginine, which supports the growth of allografted cancer cells^124^, indicating that autophagic activity in the host supports cancer cell viability^87,125^. As circulating arginine is degraded by the arginase ARG1, which is released into blood flow due to liver damage in autophagy-deficient host mice, tumorigenesis in mice with autophagy knockout in each tissue may differ from mice with systemic loss of autophagy. Alternatively, autophagy in loser cells supports their elimination during cell competition^126^, which may cause selection pressure toward an expanded population of autophagy-deficient cells in a microenvironment involving heterogeneous autophagic activity, including model mice generated by an approach involving Cre recombinase. These non-cell autonomous factors need to be taken into account to determine the effect of systemic changes in autophagy in future studies.

In summary, autophagy suppresses tumorigenesis but, once cancer has developed, it promotes tumor malignancy. Thus, to interpret the effect of autophagy deficiency in animal models and pathology, it is essential to consider the effect of autophagy defect in non-cancerous and cancer cells independently. On top of that, it should be taken to account that there exists a non-cell autonomous effect of autophagy in multiple organs and tumor microenvironment.

### Perspectives

Knowledge of the human genetics of autophagy is rapidly expanding as hereditary disorders with autophagy deficiency have been identified in the past decade, and we expect that many hereditary diseases for which other autophagy-related genes are responsible remain to be identified. We expect that the genes identified in the future will likely be biased. At present, genes encoding essential upstream regulatory factors for autophagy, such as FIP200 and ATG9, have not been associated with human hereditary diseases. This result could be partially due to functions specific to each gene, but data from cell biology have supported other possibilities. FIP200, ATG9, and the PI3K complex have essential roles in the formation of isolation membranes. However, cells deficient in the genes involved in LC3 lipidation, such as ATG5 and ATG12, show a small number of autophagosome-like structures^67^. These structures could be fused with a lysosome. Thus, it is suggested that the low residual autophagic activity observed in mice with knockout of the genes involved in LC3 lipidation could be sufficient to support embryonic development, although complete defects in autophagy result in embryonic lethality^85^. Therefore, hypomorphic alleles of the upstream factors could be identified as the cause of a hereditary disease with severe symptoms. Alternatively, similar to *becn1*^*+/−*^ mice, which exhibit a high carcinogenic phenotype^127,128^, those with heterozygous mutations with haploinsufficiency may show some aspects of autophagy deficiency.

Since the molecular mechanism of autophagy has not been fully clarified, it is predicted that there are many unidentified factors related to autophagy. In future studies, the factors related to hereditary disorders could be recognized as autophagy-related disorders. One example is optineurin (OPTN), which is involved in selective autophagy. *OPTN* is known as one of the causative genes of ALS^129^. To date, the role of OPTN in autophagy is focused on the autophagy receptor, which helps sequester ubiquitinated substrates by isolation membranes via binding to both LC3 and ubiquitin^130,131^. However, recent reports have indicated that *OPTN* knockout cells have decreased autophagosome formation induced by starvation^132^. *SQSTM1*, a gene encoding p62, is one of the best-investigated substrates for autophagy. While it functions as a receptor protein to tether LC3 with targets for selective autophagy, several studies have suggested a possible involvement of p62 in autophagosome formation itself as well^66,133^. Interestingly, homozygous mutations in *SQSTM1* were identified as a cause of human neurodegeneration with ataxia and other associated symptoms^134^. Of course, symptoms of the patients or phenotypes of model mice are expected to result from a combination of autophagy deficiency and aberrations other than autophagy, so it is requisite to carefully estimate the contribution of autophagy for suppression of each disease. To date, there are several powerful tools to estimate autophagy in vivo such as GFP-LC3 mice, GFP-LC3-RFP-LC3ΔG mice, and mt-keima mice^71,135–137^. However, it remains technically difficult to estimate autophagy activity in human. Moreover, a cavity of the model systems is that markers of autophagosome do not always necessarily indicate autophagy in vivo; LC3 is related to other events such as LC3-associated phagocytosis (LAP)^138^. Therefore, interpretation using both the animal models and the approach of cell biology is necessary to understand the role of autophagy in pathology.

The expansion of autophagy-related diseases may increase the demand for therapy. More strikingly, there are many lifestyle-related diseases with autophagic abnormalities. For example, autophagy is decreased in the fatty liver caused by excessive nutrition. This phenomenon is mainly due to the accumulation of Rubicon protein, which is a negative regulator of autophagy, and Rubicon knockout mice showed reduced hepatocyte cell death and fat accumulation associated with fatty liver^79^. In addition, autophagic activity gradually decreases as aging progresses and increased basal autophagy extends lifespan and healthspan in many organisms including mice^139–141^. Our group recently showed that the Rubicon protein level increases with aging and that Rubicon knockout reduces various diseases associated with aging^142^. In addition to the genetic diseases outlined here, determination of the pathology and molecular mechanisms of the changes in autophagic status in lifestyle-related diseases and the aging process is needed to develop novel strategies for human healthcare. In addition to basic research to elucidate the mechanisms of autophagy, further studies on these topics are needed in the future.
